# Engineering natural molecule-triggered genetic control systems for tunable gene- and cell-based therapies

**DOI:** 10.1016/j.synbio.2023.06.002

**Published:** 2023-06-12

**Authors:** Xinyi Wang, Xuantong Zhou, Liping Kang, Yuqin Lai, Haifeng Ye

**Affiliations:** Synthetic Biology and Biomedical Engineering Laboratory, Biomedical Synthetic Biology Research Center, Shanghai Key Laboratory of Regulatory Biology, Institute of Biomedical Sciences and School of Life Sciences, East China Normal University, Dongchuan Road 500, Shanghai 200241, China

**Keywords:** Mammalian synthetic biology, Natural molecules, Genetic switches, Gene- and cell-based therapy, Precision medicine

## Abstract

The ability to precisely control activities of engineered designer cells provides a novel strategy for modern precision medicine. Dynamically adjustable gene- and cell-based precision therapies are recognized as next generation medicines. However, the translation of these controllable therapeutics into clinical practice is severely hampered by the lack of safe and highly specific genetic switches controlled by triggers that are nontoxic and side-effect free. Recently, natural products derived from plants have been extensively explored as trigger molecules to control genetic switches and synthetic gene networks for multiple applications. These controlled genetic switches could be further introduced into mammalian cells to obtain synthetic designer cells for adjustable and fine tunable cell-based precision therapy. In this review, we introduce various available natural molecules that were engineered to control genetic switches for controllable transgene expression, complex logic computation, and therapeutic drug delivery to achieve precision therapy. We also discuss current challenges and prospects in translating these natural molecule-controlled genetic switches developed for biomedical applications from the laboratory to the clinic.

## 1 Introduction

Synthetic biology aims to design and create intelligent gene networks, devices, and functional organisms with novel and useful functions for biotechnological and therapeutic applications [[1], [2], [3]]. Precise genetic control systems responsive to various signal inputs are becoming increasingly required for biomedical applications, including gene editing [4], epigenetic remodeling [5], drug discovery [6], T-cell therapy [7,8], and cell-based therapies for diseases such as obesity [9], psoriasis [10], diabetes [11], and hyperuricemia [12]. In the context of precision medicine, engineered cell-based therapies are recognized as the most advanced tools [13,14], and the ability to control the expression or release of therapeutic outputs remotely is essential for the functionality of synthetic devices in translational biomedical applications [[15], [16], [17], [18]].

For the early synthetic biological designs used in basic biology research and cell-based therapies, antibiotics such as tetracycline [19], doxycycline [20], and macrolides [21] are used as routine triggers. Although these conventional gene regulation systems show excellent regulation performance *in vitro* and *in vivo*, the improper use of antibiotics may have hidden risks such as the production of drug-resistant bacteria and other side effects [22]. These limitations restrict the long-term use and wide-ranging application of these systems. To avoid the development of antibiotic resistance, researchers have exploited other kinds of chemical inducers such as hormones, hormone analogs [23], biotin [24], and prescription drugs [25], but these chemical molecules are not attractive triggers, as they might be incompatible with the diverse biological processes of mammalian cells and cause metabolic interference and physiological disorders [26]. To address these problems, an ideal trigger molecule for multiple biomedical and clinical applications is required to meet the following criteria: i) it is a side-effect-free physiologic inducer, ii) it is non-toxic at regulation-effective concentrations, iii) it consists of clinically approved components, and iv) it is inexpensive and affordable.

**Fig. 1:**
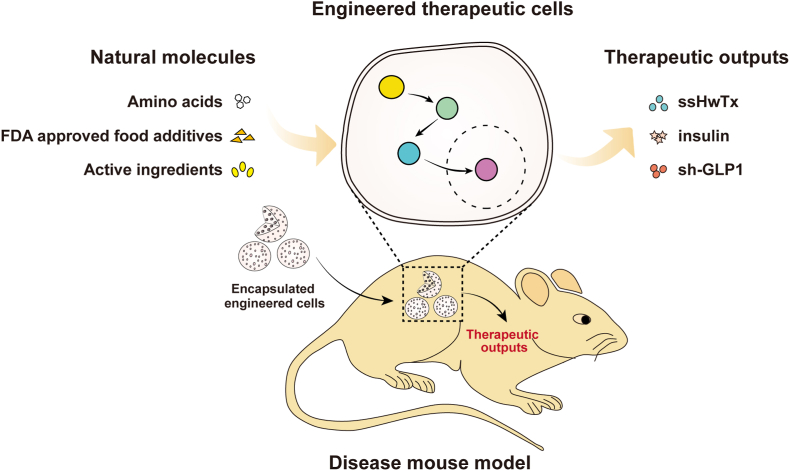
The programming of natural molecule-inducible engineered cells for therapeutic purposes. Engineered therapeutic cells have the capability to be finely regulate the expression of therapeutic proteins in response to specific natural small molecules. In a concise overview, natural molecule-responsive engineered cells are implanted into a disease mouse model. Subsequently, the administration of the corresponding natural molecules facilitates the expression of therapeutic proteins, leading to the expected therapeutic benefits.

Natural products encompass a vast array of compounds derived from nature, showcasing remarkable structural complexity, profound bioactivity, and profound evolutionary significance. Most natural products serve as sources of nutrients for bacteria [27], fungi [28], plants [29], and animal cells [30] and may promote cell proliferation and accelerate metabolism [29,31]. In addition, some natural products are natural metabolites, which widely exist in plant roots, stems, leaves, and fruits [32]. Natural products are characterized by their origin, as they are produced by biological systems, in contrast to being purely synthetic or artificially created in a laboratory. Further, natural products tend to have intricate chemical structures, often consisting of multiple rings, functional groups, and stereocenters. This complexity arises from the intricate biosynthetic processes involved in their production. Generally, natural products are characterized as soluble [33], nontoxic [34], biodegradable [35], and even beneficial to health because of their anti-infective [36], antioxidant [37], anti-inflammatory [38], and anti-cancer activities [39]. These unique features and biological activities have promoted the wide application of natural products in a variety of industries; for instance, they have been used in the development of cosmetic ingredients [40], food additives [41], and healthcare products [42]. In the field of synthetic biology natural products are rapidly developed into ideal triggers of engineered gene control systems [43]. In recent decades, natural compounds have been successfully programmed to control gene expression *in vitro* and in living animals. Natural product molecule-triggered systems have offered a new tool for treating metabolic and neurological diseases through the production of adjusted doses of therapeutic proteins. Briefly, upon administration of these specific natural molecules, the implanted engineered cells trigger the expression of therapeutic proteins, thereby providing the intended therapeutic benefits (Fig. 1).

In this review, we first list the potential sources of natural molecules that are available to control gene circuits. Then, we introduce the general design strategies for natural product-induced systems for mammalian synthetic gene networks. Next, we outline typical applications and advances in the use of natural product-controlled systems. Finally, we discuss the various challenges and future prospects for the use of natural product-controlled cells in synthetic biology.

## 2 The sources of natural molecule triggers

Synthetic gene circuits for future cell-based therapies and the biopharmaceutical manufacturing industry require non-toxic, safe, and adjustable inducers, which exhibit minimal side effects, maximal efficiency, and optimal pharmacokinetics in terms of their absorption, distribution, metabolism, and excretion within mammalian organisms [[44], [45], [46]]. Developing genetic switches that can be controlled by small biochemical molecules derived from natural products is in high demand. There are four main sources of eligible natural inducers: i) Amino acids, the basic structural units of biomolecules, are a significant source of side-effect-free physiologic inducer molecules. Some early studies used amino acids such as l-arginine [47] and tryptophan [48] to regulate gene switches. ii) FDA approved food additives and natural cosmetics additives. For example, phloretin [49], vanillic acid [50], benzoic acid [51], and menthol [52] have been developed to control synthetic gene switches. iii) Active ingredients derived from drinks, functional foods, and fruits that are consumed daily. For example, caffeine [53] and protocatechuic acid (PCA) extracted from green tea [54] have been used to regulate gene switches. iv) Medical or health care products with specific pharmacological actions. For example, oleanolic acid (a typical over-the-counter [OTC] liver protection drug) [55], ursolic acid (an effective anti-cancer drug) [56], and ferulic acid (an antithrombotic drug) have been utilized to regulate synthetic gene circuits. The notable advantage of using these natural molecules as a regulatory medium is that, while effectively regulating the expression of functional genes, they can also have excellent biological activity, and thus realize double or multiple therapeutic efficacies, especially for the treatment of cardiovascular disease, hepatogenous diabetes, obesity, and other complex metabolic diseases [14].

## 3 Three different gene switch design strategies

The rapid development of basic research has enabled scientists to gain a better understanding of the biomolecular mechanisms of life activities. Synthetic biologists are devoted to exploiting physiological molecular components and rationally designing, transforming, reconstructing, and modularizing them for biotechnological and therapeutic applications [57]. These inducible genetic switches can be generally divided into three distinct groups based on different design strategies. One is a heterogenous regulation system composed of xenogeneic regulatory components obtained from prokaryotic bacterial systems and eukaryotic gene expression elements [58]. Another design strategy revolves gene circuits that are designed by combining receptor-based methods with endogenous signaling pathways in mammalian cells [59]. A third approach entails the utilization of dimerization-based strategies, wherein two distinct protein domains or subunits are engineered to interact and form a functional complex upon activation by an inducer. By fusing these protein domains or subunits with transcriptional regulators, scientists can create gene switches that are activated upon dimerization in response to naturally occurring molecules.

### 3.1 Gene switches designed using prokaryotic components

To date, several heterologous transcription control modalities in mammalian cells and transgenic animals have been described. The prevailing design consists of a heterologous synthetic transactivator (transrepressor) assembled by fusing a prokaryotic response regulator to a transactivation (or transrepression) domain, and a chimeric transactivator-specific promoter engineered by integrating a prokaryotic operator into a minimal/constitutive eukaryotic promoter [47,49,58]. Such basic gene switches either induce (ON-type systems) or repress (OFF-type systems) transcription of linked transgenes [50,60,61]. Gene transcription can be regulated by a specific inducer, which modulates the promoter affinity of chimeric transcription factors in a dose-dependent manner. By optimizing the number of copies of operators, replacing different transactivator variants, redesigning the chimeric promoter, and testing functions in different mammalian cell lines, adjustable and reversible transcription control of the specific target gene can be achieved.

**Fig. 2:**
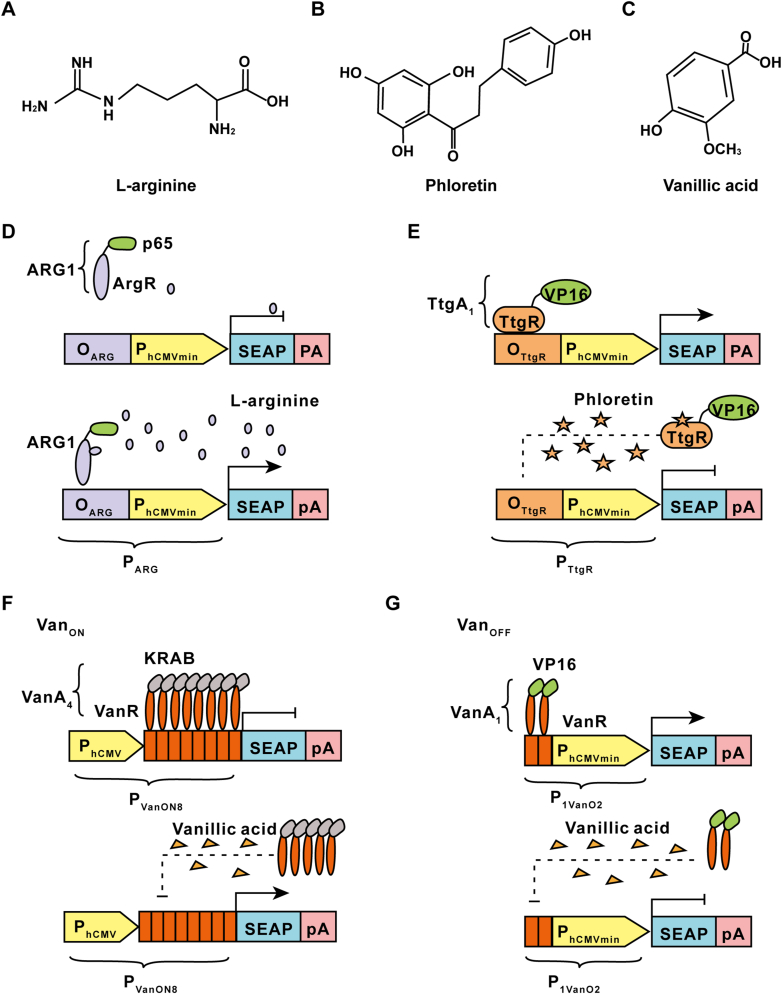
Natural molecular triggers-mediated gene regulation. (A–C) Chemical structures of the representative natural molecules, including l-arginine (A), phloretin (B), vanillic acid (C). (D) The bacterial repressor of *Chlamydia pneumoniae* ArgR fused with a transactivation domain of NF-kB p65 was constitutively expressed under the control of the simian virus 40 promoter. In the presence of l-arginine, the hybrid arginine-responsive transactivator (ARG_1_) will specifically bind to l-arginine-responsive promoter (P_ARG_), including the ARG operator O_ARG_ and the minimal version of the cytomegalovirus immediate early promoter P_hCMVmin_ and drive expression of human placental secreted alkaline phosphatase (SEAP). (E) The *P. putida* DOT-T1E-derived bacterial repressor TtgR was fused to the VP16 transactivation domain of *H. simplex virus* to create a constitutively expressed transactivator TtgA_1_ (TtgR-VP16). In the absence of phloretin, TtgA_1_ binds to the phloretin-responsive promoter (P_TtgR1_), including a phloretin operator O_TtgR_ and the minimal version of the cytomegalovirus immediate early promoter P_hCMVmin_, inducing SEAP expression. In the presence of phloretin, TtgA_1_ is released from P_TtgR1_, which switches SEAP expression off. (F) For the VAN_ON_ system, transcriptional repressor VanR is fused to the KRAB transrepressor domain, a human Krueppel-associated box protein, generating a constitutively expressed VanA_4_ (VanR-KRAB). In the absence of vanillic acid, VanA_4_ binds to a chimeric target promoter P_VANON8_, including the VAN operator human cytomegalovirus immediate early promoter P_hCMV_ and represses SEAP expression. In the presence of vanillic acid, VanA_4_ is released from P_VanON8_, which fully induces SEAP expression. (G) For the VAN_OFF_ system, VanR is fused to the VP16 transactivator domain of the *Herpes simplex virus*, resulting in constitutively expressed VanA_1_ (VanR-VP16). In the absence of vanillic acid, VanA_1_ binds to a chimeric target promoter P_1VanO2_, including the VAN operator O_van_ and the minimal version of the cytomegalovirus immediate early promoter P_hCMVmin_ and drives SEAP expression. In the presence of vanillic acid, VanA_1_ is released from P_1VanO2_, which represses SEAP expression.

The first and exemplary design of a gene switch activated by a natural molecule was developed by Hartenbach et al., in 2007 [47]. In their study, the research team engineered an arginine sensor derived from the human pathogen *Chlamydia pneumoniae* [62] which facilitated arginine-adjustable transcription (ART) control in both mammalian cells and mice (Fig. 2A and D). They engineered a hybrid arginine-responsive transactivator (ARG) by fusing P65 (a transactivation domain of NF-kB) to the bacterial repressor ArgR, and engineered an arginine-responsive promoter (P_ART1_) harboring an ArgR-specific operator sequence (O_ARG_) followed by a minimal version of the cytomegalovirus immediate early promoter (P_hCMVmin_). In the presence of arginine, the ARG specifically binds O_ARG_ then initiates target gene expression. As an ON-type system, the bacterial repressor ART system remains silent under endogenous l-arginine concentrations but can be triggered by increasing l-arginine levels.

In 2009, Gitzinger et al. employed the flavonoid-triggered repressor TtgR operon derived from *Pseudomonas putida* (DOT-T1E) to develop a mammalian phloretin-adjustable control element (PEACE) system. This innovative system allowed for finely controlled transgene expression in various mammalian cell lines (Fig. 2B and E) [49]. Upon the application of phloretin-containing skin lotions in appropriate quantities to the skin of mice implanted with microencapsulated cells harboring the PEACE system, the induction of target gene expression occurred. Consequently, this led to the modulation of heterologous protein levels in the bloodstream. The utilization of the PEACE system significantly broadened the scope of phloretin in biomedical applications, which had primarily been limited to its use as a cosmetic additive due to its antioxidant properties [63].

Another noteworthy example is the vanillic acid induction system developed by the same research group [50]. Vanillic acid (Fig. 2C) is a licensed food additive derivative that serves as a flavoring agent [64]. Researchers have engineered different variants of the synthetic vanillic acid-responsive mammalian expression systems (VAC), including the VAC_ON_ system (Fig. 2F) and the VAC_OFF_ system (Fig. 2G), by utilizing the vanillic acid-responsive transcriptional repressor VanR from *Caulobacter crescentus*. These one-input and one-output transgenic switches have demonstrated efficacy in controlling the expression of reporter genes in stable transgenic mammalian cell lines. Additionally, when mice were transplanted with these customized cells, their serum secreted alkaline phosphatase (SEAP) levels could be modulated upon treatment with vanillic acid [50].

Prokaryotic trigger-regulatory repressors, capable of modulating their operator-binding affinity in response to specific designated molecules, have provided a consistent foundation for the development of orthogonal mammalian transcription-control devices [60,65,66]. However, certain preliminary transgenic devices possess limitations, such as a singular regulation mode, which hampers their ability to perform more complex regulatory tasks [47,49]. Additionally, their restricted adjustable range often renders them ineffective for achieving desired biological effects in practical applications [47,60]. It is noteworthy that certain systems exhibit poor specificity, as they can be activated by molecules belonging to the same category, thereby increasing the risk of non-specific activation or inhibition. For instance, various flavonoids can activate the phloretin-regulated system to varying extents [49]. Thus, to enhance specificity for practical translational applications, further optimization is imperative.

### 3.2 Gene switches designed through receptor-based methods

Because gene circuits built using prokaryotic response regulators have possible side effects and may induce negative immune responses [[67], [68], [69]], researchers have worked to develop gene switches that employ receptor-based methods by combining human components in recent years.

For example, in 2017, Xue et al. engineered an oleanolic acid (OA)-triggered gene expression device for treating hepatogenous diabetes by combining the recently discovered OA, which is a strong GPBAR1 (a G-protein-coupled receptor) agonist, with the human cyclic AMP (cAMP)-related signaling pathway [55]. In 2018, Bojar et al. used a caffeine-binding single domain antibody fused with the EpoR receptor linked to the JAK/STAT3 pathway to construct a sensitive transgenic switch, which regulated the expression of downstream Glucagon like peptide-1 (GLP-1) in the treatment of type 2 diabetes in mice [53]. In 2019, inspired by the human ion channel receptor (TRPM8) sensing pathway, which can be stimulated by menthol, Bai et al. developed a menthol-triggered orthogonal transgene switch to regulate the production of therapeutic proteins for the treatment of hyperglycemia and muscle atrophy [52].

Conventional prokaryotic gene switches featuring well-tuned heterologous components in mammalian cells, which were developed earlier, showed the ability to potently and sensitively regulate genes for disease therapeutic intervention [15], but such devices have the potential for biological incompatibility in practical clinical application [17]. Therefore, more and more synthetic biologists have tried to capture human physiological signals that can be safely converted into fine-tuned therapeutic uses. Some pioneering studies have shown the great potential of synthetic gene circuits modified using native human sensors with high ligand specificity such as G-protein-coupled receptors for future clinical applications [9,52,55,70]. However, these sensors may cause signal interference with the endogenous system, and the sensitivity and adjustable range of these sensors may be limited, as it is hard to reconstruct endogenous pathways [9,59]. To sum up, each of these two design strategies has its own advantages and limitations. Therefore, researchers can select different strategies according to the desired natural molecule-associated sensing pathway and the actual application requirements.

### 3.3 Gene switches designed employing the dimerization strategy

The utilization of natural molecules-induced self-dimerization or heterodimerization has gained considerable popularity as an effective design strategy within the realm of transcriptional regulation in synthetic biology. This approach entails the fusion of dimerization proteins with artificial transcription activation and DNA-binding domains (DBDs). Through the application of small molecules to induce homodimerization or heterodimerization, transcriptional complexes are formed, subsequently activating gene expression.

The pioneering work of Pollock et al., in 2002 established the most iconic design of a gene switch induced by natural molecules, utilizing the dimerization strategy [71]. They achieved remarkable success in engineering a rapamycin-induced dimerization system involving two proteins, FK506-binding protein (FKBP), and the rapamycin-binding domain of FRAP (FKBP rapamycin-associated protein). In the presence of rapamycin, these proteins can form a heterodimer. To be specific, FRAP is fused to the S3H activation domain, resulting in the creation of a rapamycin-dependent transactivator, while the zinc-finger DNA-binding domain is fused to FKBP to generate a fusion rapamycin sensor domain. When rapamycin is present, it induces the dimerization of the transactivator and fusion rapamycin sensor domain, causing the combined protein complex to translocate into the nucleus and initiate transgene expression. Through the use of this rapamycin-mediated dimerization system, they successfully activated endogenous *VEGF* gene expression by incorporating a zinc-finger DNA-binding domain that targets the chromosomal human *VEGF* gene. By manipulating the configuration of fusion proteins and FKBP/FRAP, researchers have developed a diverse range of rapamycin-induced devices for controlling gene expression, protein-protein interactions, and therapeutic interventions for diseases. However, it is important to note that *in vivo* applications of this system are limited due to the presence of abundant endogenous mammalian FKBP, and rapamycin may induce adverse effects by interfering with the endogenous mammalian target of rapamycin (mTOR) pathway.

In 2011, Liang et al. introduced a novel approach to address the aforementioned concerns by developing an abscisic acid (ABA)-induced proximity system for gene expression regulation [72]. ABA, a cost-effective, non-toxic, and readily available plant hormone, was utilized to induce the dimerization of ABI/PYL1. In their strategy, they engineered an ABA-activator cassette by fusing the yeast Gal 4 DNA binding domain (Gal4DBD) to ABI and the herpes simplex virus VP16 transactivation domain (VP16AD) to PYL. By leveraging ABA, they facilitated the proximity of the transactivation complex (Gal4DBD and VP16AD), resulting in robust activation of gene transcription. Their research successfully demonstrated ABA-induced gene transcription, protein subcellular localization, and signal transduction in cultured mammalian cells. This innovative system holds great potential for regulating gene expression by employing ABA as a highly effective inducer.

In addition, Gao et al. introduced a dCas9-based platform that exploits both abscisic acid (ABA) and gibberellin (GA) to achieve highly efficient gene activation and repression in mammalian cells [73]. They capitalized on the dimerization properties of abscisic acid (ABA)-inducible ABI/PYL1 [72] and gibberellin (GA)-inducible GID1/GAI to develop their dCas9-based platform for efficient gene activation in mammalian cells [74]. In this approach, dCas9 and a transcriptional effector were fused to complementary pairs of heterodimerization domains (ABI/PYL1 or GID1/GAI), which only assembled in the presence of specific inducers. The researchers designed a range of logic operators, including AND, OR, NAND, NOR, as well as a diametric regulator that activated gene expression with one inducer and repressed it with another. This platform showcases the versatility of employing ABA and GA as external triggers to precisely control gene expression in mammalian cells.

In summary, the utilization of small molecule-induced dimerization systems in gene regulation has gained significant popularity and presents tremendous potential for the advancement of synthetic biology tools. These systems are highly valued for their simplicity and modularity, enabling precise control over gene expression. With continuous progress in this field, we anticipate the emergence of additional tools that harness the benefits of small molecule-induced dimerization, thereby further expanding our capabilities for precise gene regulation.

## 4 Applications

### 4.1 Spearmint-engineered cells for chronic pain treatment

Chronic pain is one of the most difficult neurological diseases to manage [75,76]. Current treatment options for chronic pain are often restricted by dose-limiting toxicities, drug tolerance, and even risk of addiction [77,78]. Therefore, there is an urgent need for new therapeutic approaches with high safety and efficiency. In 2018, Wang et al. exploited a novel pain management strategy based on cell-engineering principles and a natural molecule-triggered gene switch, which successfully enabled long-term management of chronic pain through oral administration or inhalation of spearmint [79]. Spearmint is derived from an edible natural plant and is used as a food flavoring agent, which has been added to a wide range of medicines, ointment beauty products, and food products [80,81]. Spearmint essential oil is considered an ideal trigger for control of gene transcription because it is safe and inexpensive. In this study, researchers reconstructed a spearmint-stimulated transcription control system in a human embryonic kidney cell line (Hana3A), which they called AromaCell, and successfully applied it to reduce chronic inflammation and neuropathic pain in mouse models.

**Fig. 3:**
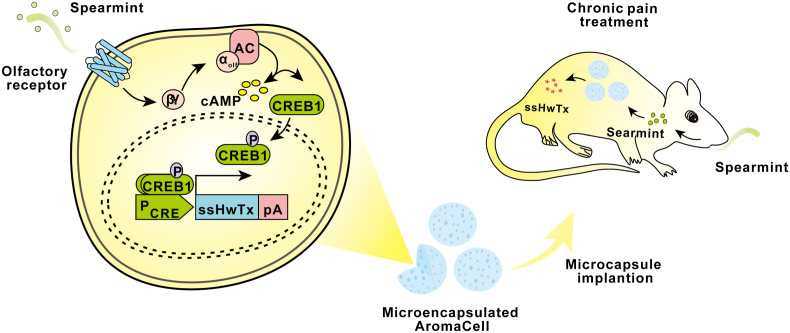
Spearmint-engineered cells for chronic pain treatment. Upon treatment with spearmint, the constitutively expressed R-carvone-specific mammalian olfactory receptor (OR) is induced and activates adenylate cyclase (AC), which triggers the cAMP signal pathway, causing phosphorylation of the human transcriptional factor CRE binding protein 1 (CREB1) and its nuclear translocation. Nuclear-localized CREB1 activates the CRE promoter to express the downstream gene ssHwTx. Human embryonic kidney cells (Hana3A) stably expressing the spearmint aroma R-carvone-stimulated ssHwTx (AromaCells) were microencapsulated into alginate-poly-(l-lysine)-alginate beads, allowing free diffusion of nutrients and secreted proteins across the semipermeable membrane while simultaneously shielding encapsulated cells from the immune system. After oral intake or inhalation-based intake of spearmint essential oil, the implanted microcapsules containing AromaCells secrete protein therapeutics into the bloodstream of a chronic inflammatory and neuropathic pain mouse model.

An illustration of the AromaCell system is shown in Fig. 3. Spearmint-triggered olfactory neuron-specific G protein mediates activation of adenylate cyclase via ectopic expression of R-carvone-specific mammalian olfactory receptor and receptor transporter 1 (RTP1) in human cells. Then the cAMP signal pathway is activated, which causes phosphorylation of the transcriptional factor cAMP-responsive binding protein 1 (CREB1) and its nuclear translocation, thus activating the CRE promoter and expression of downstream genes, and ultimately, production of the secretion-engineered and stabilized HwTx-IV (ssHwTx, a safe and potent analgesic peptide that selectively inhibits the pain-triggering voltage-gated sodium channel NaV1.7). Microencapsulated AromaCells were implanted into a chronic inflammatory and neuropathic pain mouse model, and ssHwTx production in these mice could be precisely controlled by spearmint aromatherapy. The mice showed a reduction in pain-related behaviors after oral administration or inhalation of spearmint essential oil, without cardiovascular, immunogenic, or behavioral side effects.

This device utilized the non-invasive nature of the spearmint aroma compound to engineer human cells, which provided a robust, tunable, and on-demand therapeutic protein secretion system for treating chronic pain. This new customized cell therapy strategy, aroma induction, which avoids side effects caused by excessive drug toxicity, might become the therapy of choice for life-long management of chronic pain.

### 4.2 Vanillic acid triggers human iPSCs to differentiate into islet β-like cells

Stem cell research has shown that cell-fate decisions are adjusted by various mechanisms, including morphogen gradients, lateral inhibition, microRNAs, epigenetic modifications, and regulated activation and silencing of crucial transcription factors [[82], [83], [84]]. Synthetic biology studies have offered a natural toolbox of biological building blocks and gene network topologies for the construction of synthetic lineage-control networks, which can program cell fate [84,85]. Compared with approaches using growth-factor or chemical-based cocktails for stem cell reprogramming, synthetic lineage-control networks are expected to be more economical and to enable trigger-programmable simultaneous control of ectopic and chromosomally encoded transcription factor variants with precise differential transcription factor expression switches.

**Fig. 4:**
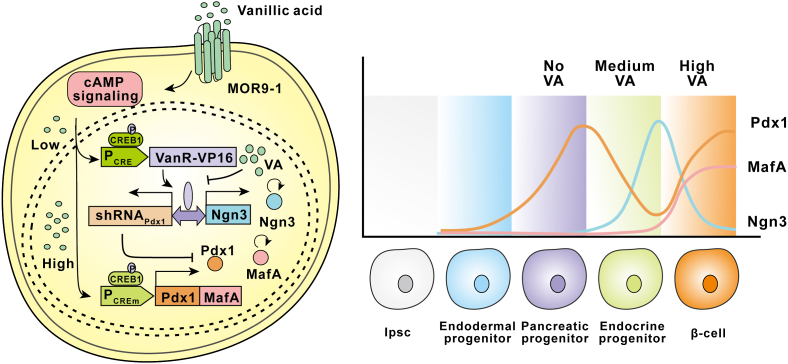
Vanillic acid triggers human iPSCs to differentiate into islet β-like cells. The G-protein-coupled receptor MOR9-1, which is responsive to vanillic acid, is constitutively expressed. Under medium vanillic acid concentrations, the human transcriptional factor CRE binding protein 1 CREB1 responsive promoter (P_CRE_) drives the expression of VanR-VP16, which binds to and activates the bidirectional vanillic acid-responsive promoter and initiates the expression of codon-modified Neurogenin 3 (Ngn3) and pdx1-shRNA. Consequently, Ngn3 levels switch from low to high (OFF-to-ON), and Pdx1g levels toggle from high to low (ON-to-OFF). At high vanillic acid levels, VanR-VP16 is released from its promoter, and both Ngn3cm and pdx1g-shRNA are shut down. At the same time, the lower-sensitivity promoter (P_CREm_) drives the co-cistronic expression of Pdx1 and MafA. Overall, the mutually exclusive expression switches for Ngn3 (OFF-ON-OFF) and Pdx1 (ON-OFF-ON) as well as the concomitant induction of MafA (OFF-ON) expression ensure the differentiation of human iPSCs to β-like cells.

In 2016, Martin Fussenegger's group skillfully developed a vanillic acid-triggered synthetic lineage-control network by assembling synthetic signaling cascade- and transcription factor-based gene switches [86]. By taking advantage of a gene switch based on a negative regulatory operon sensitive to vanillic acid in bacteria [50], this group designed a synthetic lineage-control network consisting of vanillic acid-triggered mutually exclusive switches for the expression of three transcription factors, neurogenin 3 (Ngn3), pancreatic and duodenal homeobox 1 (Pdx1), and V-maf musculoaponeurotic fibrosarcoma oncogene homolog A (MafA) (Fig. 4). This regulatory network enables the differentiation of human induced pluripotent stem cell (hiPSC)-derived pancreatic progenitor cells into glucose-sensitive insulin-secreting β-like cells, whose glucose-stimulated insulin-release dynamics compare favorably with those of human pancreatic islet cells.

When stimulated by vanillic acid at medium concentrations, a G protein-coupled receptor activates the cAMP signaling pathway, and the CREB1 -sensitive promoter driving VanR-VP16 expression plays the dominant role, which then induces expression of Ngn3 and Pdx1_shRNA_. Consequently, Ngn3 levels switch from low to high (OFF-to-ON), and Pdx1g levels toggle from high to low (ON-to-OFF). When stimulated by a high concentration vanillic acid, VanR-VP16 is released from its promoter, which turns off the expression of Ngn3 (OFF-ON-OFF) and shRNA. By contrast, Pdx1 (ON-OFF-ON) and MafA (OFF-ON) are abundantly expressed. In this way, Pdx1, Ngn3, and MafA can be precisely regulated and expressed in different network topologies, which enables the differentiation of human iPSCs into artificial glucose-sensitive insulin-secreting β-like cells [86].

This work showed that trigger-adjustable basic transgene-controlled devices are compatible and can be assembled into higher order control networks. In addition, it indicated that synthetic lineage-control networks may serve as a new design blueprint for genetically reprogramming somatic cells into autologous cell phenotypes for regenerative medicine.

### 4.3 Natural molecule-engineered mammalian cells in diabetes treatment

Diabetes is one of the most severe of the metabolic diseases that seriously affect human health [87]. Traditional methods of diabetes treatment have many deficiencies and limitations, such as the inconvenience of repeated insulin injection and side effects of oral chemical drugs [88,89]. Cell therapy, in which the expression of insulin or GLP-1 is accurately controlled *in vivo*, provides a novel strategy for diabetes treatment.

#### 4.3.1 OA-controlled functional cells in treatment of hepatogenous diabetes

**Fig. 5:**
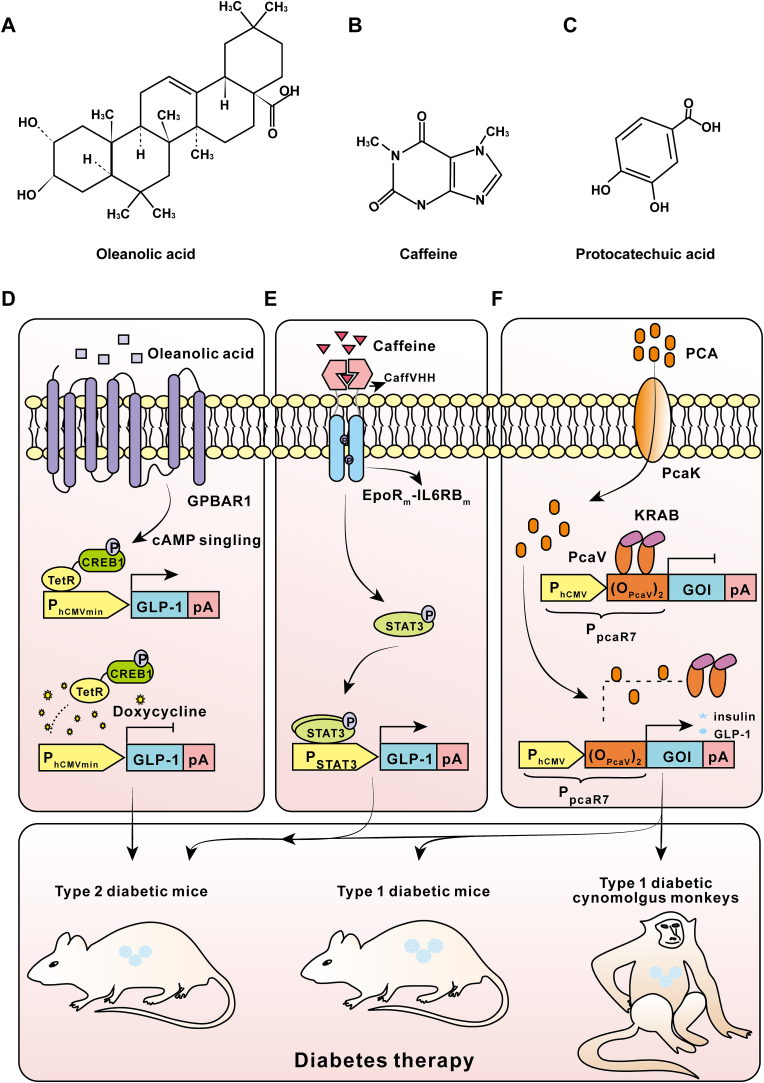
Natural molecule trigger-engineered mammalian cells in diabetes treatment. (A–C) Chemical structures of the representative natural molecules, including oleanolic acid (A), caffeine (B), and protocatechuic acid (C). (B) Oleanolic acid-inducible gene circuits for hepatogenous diabetes therapy. Oleanolic acid activates ectopically expressed human G-protein-coupled bile acid receptor 1 (GPBAR1), which triggers the cAMP signaling pathway, phosphorylating transcriptional factor CREB1. The phosphorylated TetR-CREB1 fusion protein transactivates minimal inducible promoters containing TetR-specific operator sites and induces GLP-1 expression. In the presence of doxycycline, TetR-CREB1 is released from its operator, which switches SEAP expression off. HEK293 cells, which are stably transgenic for oleanolic acid-inducible gene circuits, are microencapsulated into alginate-poly-(l-lysine)-alginate and implanted into type 2 diabetic mice. (C) Caffeine induces homodimerization of the aCaffVHH-EpoRm-IL-6RBm hybrid protein, which leads to phosphorylation of STAT3 by JAK kinases. The STAT3-responsive promoter P_STAT3_ is then activated, leading to transcriptional activation of the downstream gene *GLP-1*. HEK293 cells, which are stably transgenic for the oleanolic acid-inducible GLP-1 system, are microencapsulated into alginate-poly-(l-lysine)-alginate and implanted into diabetic mice for type 2 diabetic therapy. (D) The synthetic mammalian PCA-triggered transrepressor PcaR (KRAB-PcaV) is an N-terminal fusion of PcaV with a *trans*-silencing Krueppel-associated box (KRAB) domain. In the absence of PCA, PcaR binds to a chimeric target promoter, P_PcaR7_, and represses therapeutic protein expression; in the presence of PCA, PcaR is released from P_PcaR7_ and activates GLP-1 or insulin expression. PcaK is the transporter of PCA; with the help of PcaK, PCA molecules are easily pumped from the culture medium into cells, which results in the release of PcaV-KRAB from P_PcaR7_ and the initiation of GLP-1 or insulin expression at a relatively low PCA concentration. PCA-triggered HEK_PCA-ON-GLP-1/insulin_ cells are microencapsulated and implanted into type 1 and type 2 diabetic mice as well as type 1 diabetic cynomolgus monkeys for diabetic therapy.

In 2017, Xue et al. used natural product-engineered designer cells to correct type 2 diabetes [55]. Specifically, they constructed an OA-induced therapeutic gene device. OA is a non-toxic plant-derived triterpenoid compound with antitumor, antiviral, and hepatoprotective properties, which is widely distributed in plant foods and products such as olive oil, olive leaves, apples, and red beets [56]. In Chinese pharmacies, commercial OA tablets are available as OTC drugs for treating acute and chronic hepatitis infections [90]. Researchers ectopically expressed human G protein coupled receptor 1 (GPBAR1) [91]. When induced by OA, GPBAR1 activates adenylate cyclase, which converts ATP into cAMP. After the binding of cAMP with the regulatory subunit of protein kinase A (PKA), the catalytic subunit of PKA enters the nucleus and phosphorylates CREB1. The phosphorylated TetR-CREB1 complex then recognizes TetO_7_ and activates the expression of GLP-1. Furthermore, in presence of doxycycline (Fig. 5A), the TetR-domain is unable to bind TetO_7_, resulting in the immediate termination of transgene expression, which provides an increased capacity for control of unforeseeable scenarios *in vivo* (Fig. 5D). The researchers then implanted biocompatible microencapsulated transgenic HEK293 cells containing an OA-inducible Dox-repressible short human GLP-1 (shGLP-1) expression device into a hepatogenous diabetic mouse model. OA triggered accumulation of shGLP-1 in the bloodstream within 48 h, which was sufficient for substantial restoration of the animals’ glycemic control and insulin sensitivity. Remarkably, this cell implantation therapy also rapidly attenuated the most critical hepatogenous diabetes symptoms, including insulin resistance, dyslipidemia, and excessive liver enzyme activity, whereas the use of OA tablets or shGLP-1 alone failed to achieve the desired therapeutic effect. This work showed that OA not only plays a protective role in liver, but also acts as an effective natural inducer to control therapeutic protein expression for hepatogenous diabetes treatment, which relieves the burden of liver metabolism by general multi-drug treatment.

#### 4.3.2 Caffeine-inducible gene switches in the treatment of type-2 diabetic mice

In 2018, Bojar et al. designed a coffee-induced synthetic transcription control system (C-STAR) [53]. Caffeine, universally known as a central nervous stimulant that temporarily dispels sleepiness, is a xanthine alkaloid compound mainly occurring in coffee beans and tea [92]. They engineered coffee-induced designer cells bearing the C-STAR system to treat obesity-induced type 2 diabetic mouse models and achieved an excellent curative effect. In this research, scientists first engineered a fully synthetic caffeine-inducible protein dimerization system with user-defined sensitivity and functionality by utilizing a caffeine-binding single-domain VHH camelid antibody (referred to as a CaffVHH) [93], which possessed high affinity and homodimerized in the presence of caffeine (Fig. 5B). They then created various types of gene switches by fusing CaffVHH to the intracellular signaling domains of different mammalian receptor types and reconstituted synthetic signaling cascades. Caffeine-dependent STAT3-signaling was found to be the best fit in terms of potency and sensitivity to physiological caffeine levels, so it was used for cell therapy. The optimal C-STAR system is based on the Epo receptor and the JAK/STAT3 pathway (Fig. 5E).

The customized HEK-293T cells that contain the stably integrated optimal C-STAR system were found to be responsive to physiologically relevant concentrations of caffeine and exhibited precisely controlled transgene expression (SEAP reporter and a therapeutic protein such as shGLP-1). In addition, they implanted these customized cells in obesity-induced type 2 diabetic mice and verified its curative effect by measuring the expression level of shGLP-1. Compared with untreated mice, the glucose homeostasis was significantly improved after intake of certain caffeinated beverages such as tea and coffee for 2 weeks. This system, in which diabetes treatment is combined with daily lifestyle choices, may improve the compliance of patients. However, there are also some deficiencies and limitations in this system; for example, the transgene protein levels are lower compared with those of other similar systems. In addition, its orthogonality is limited by the endogenous JAK/STAT3 pathway. Moreover, this system is not specific enough, as black tea and energy drinks can also induce the induction of the transgene [53].

#### 4.3.3 A green tea-triggered genetic device for diabetes treatment in mice and monkeys

Green tea is a well-known beverage that contains abundant tea polyphenols [94]. After green tea intake, tea polyphenols are rapidly metabolized into protocatechuic acid (PCA) [95], which is reported to possess various biological activities including antioxidant, antimicrobial, and anti-inflammatory activities [96]. Because of the excellent bioactivity of PCA, in 2019, Yin et al. constructed a PCA-triggered transgene expression platform inspired by prokaryotic trigger-adjustable repressors and applied it to gene editing, biocomputing, and diabetes treatment [54].

In this study, Yin et al. engineered PCA_ON_ and PCA_OFF_ switches based on a synthetic mammalian transrepressor called PcaR, which consists of the *Streptomyces coelicolor*-derived transcriptional repressor PcaV and eukaryotic epigenetic effector domains (Krueppel-associated box [KRAB] or VP16). In the absence of PCA (Fig. 5C), PcaR (KRAB-PcaV) silences constitutive gene expression from the synthetic promoter P_PcaR_. When PCA is present, it disrupts the PcaR-dependent repression, PcaR is released from P_PcaR_, and reporter gene (SEAP) expression is induced. By optimizing the number of operator copies, the constitutive promoter, and the mammalian cell line, optimal PCA_ON_ and PCA_OFF_ switches with long-term dose-dependent, time course-dependent, and reversible induction kinetics *in vitro* and *in vivo* can be obtained. To improve the sensitivity of this system, they developed an enhanced sensitive PCA_ON_ switch by introducing the PCA transporter PcaK (Fig. 5F). When designer cells containing the PCA_ON_ switch were applied to treat type 1 and type 2 diabetic mice and nonhuman primates, a sustainable level of insulin expression was detected and glucose homeostasis was achieved via low-dose oral PCA intake or by drinking green tea for up to 15 days [54].

### 4.4 Natural molecule-mediated biocomputing *in vivo*

Several studies have shown that the behavior of cells, similar to that of electronic circuits, is regulated by sophisticated information-processing systems, which enables the cells to dynamically integrate and respond to distinct input signals [15]. Using molecular biology and bioengineering approaches, synthetic biologists have developed biological computers, which can sense different kinds of input signals and respond with corresponding instructions through accurate calculation; these biological computers can potentially be used to execute predictable metabolic and therapeutic functions [14,17,57]. The rapid development of natural molecule-triggered gene switches has enabled the development of mammalian cells that can perform a variety of complex logic operations. For example, in 2012 Professor Martin Fussenegger's team synthesized a variety of dual input logic operations that respond to phloretin and erythromycin in mammalian cells [97], and in 2018 successfully constructed a three-input two-output full adder triggered by tetracycline, phloretin, and vanillic acid [98]. However, the abovementioned logic gates still have some problems with biocompatibility and the toxicity of antibiotic-based trigger compounds. Yin et al. built the first biocomputation platform controlled by PCA and vanillic acid [54]. They constructed a variety of logic operations regulated by phenolic acid in HEK293 cells, including A NIMPLY B, B NIMPLY A, AND, OR, and NOR logic gates. Additionally, they transplanted these logic gates into mice, and validated all the gates' functions. The pioneering work of Yin et al. demonstrated the great potential of natural molecules in controlling a variety of different circuit architectures.

Another good example of natural molecule-controlled biocomputing is a system developed by Wang et al.: a biocomputing platform using the safe and clinically approved molecules ferulic acid and food additive benzoate [99]. They constructed five logic gates, namely AND, NAND, NOR, A NIMPLY B, and B NIMPLY A to achieve a variety of Boolean operations controlled by ferulic acid and benzoate in HEK293 cells. These proof-of-concept results show that complex genetic programs could be precisely controlled by natural products, indicating the potential for application of complex engineered cell-based therapeutic dosing regimens in biocomputing.

## 5 Challenges and perspectives

As described in this review, there has been substantial progress in the use of natural products in the development of gene switches, which has provided novel strategies for synthetic biology applications including remote control of gene expression, complex protein logic computations, and treatment of neuropathic pain and metabolic disease. Demonstration of the therapeutic functions of these devices confirmed that natural molecule-activated switches are able to precisely regulate the dosage of therapeutic proteins and that these switches have great potential for future basic research and clinical applications. However, several challenges need to be considered carefully before these natural molecule-controlled devices can be successfully applied in clinical practice. Firstly, the natural molecules should be completely nontoxic with high safety and able to easily enter specific cells and tissues. Secondly, these nontoxic trigger molecules must have an appropriate half-life to ensure that a single injection or oral dose can have good bioactivity within a proper time period but not lead to excessive therapeutic protein production over time. Therefore, more natural molecules that meet these requirements need to be screened for in future studies. Thirdly, although recent studies have successfully induced the production of therapeutic proteins, a high concentration of the inducer is still required. To achieve rapid response and high sensitivity, it is useful for researchers to seek appropriate transporters, which will help the natural inducers to be easily pumped into the cells of interest. Additionally, the dosage control of the transgene-encoded protein is critical when it comes to clinical therapies, but unlike optogenetic tools, many natural molecules are difficult to remove immediately once the system is activated. Thus, a feedback strategy should be considered, which can provide intelligent on and off control of gene expression. Furthermore, to improve the stability of switchable gene devices, concise programming achieved by reducing the number of circuit components will be helpful.

Most developed synthetic gene switches that are triggered by natural molecules are used for cell-based therapy. Three major challenges must be addressed before these proof-of-concept cell-based devices can be truly applied in clinical practice: identifying safe and efficient cells for engineering, developing a harmless implantation strategy, and increasing the long-term viability and activity of designer cells. To date almost all advanced gene switches have been implemented in cell lines such as HEK293 because they are easy to engineer and can effectively produce enough therapeutic substance. However, these xenogeneic immortalized cell lines may cause immune system attack and introduce the risk of tumor production [100]. To reduce the side effects and risks of engineered cells containing synthetic circuits, researchers have used microencapsulation technology to protect cells from immunity attack and at the same time ensure that cells can freely communicate with the outside [101,102]. However, several studies have demonstrated that although the immune system does not attack the implanted designer cells inside the microencapsulation, fibrosis responses and severe inflammatory reactions occur in the area surrounding the implantation site, mainly because these devices are often made of materials that can induce inflammation [103,104]. To solve this problem, scientists have developed crystallized drug formulations that inhibit inflammation reactions, but this complicates capsule manufacturing and may have unexpected impacts on the performance of gene switches [105]. In addition, the lifespan of designer cells in microencapsulation is limited, which restricts the use of synthetic gene switches for long-term cell therapy. Therefore, there is an urgent need for further improvement of encapsulation technology. Remarkably, the ability to engineer iPSCs or primary cells that are isolated from patients may provide novel solutions to minimize the above challenges related to immunocompatibility and noncarcinogenicity [106].

## 6 Conclusion and future directions

So far, the three design strategies for natural molecule-controlled systems, heterologous synthetic transactivators from prokaryotes, rewired endogenous signaling pathways from mammalian cells, and gene switches using protein dimerization methods have enabled the flexible and tunable control of protein level and activity and provided a safe and effective way to treat diseases. The multiple design strategies and the increasing number of inducible tools derived from natural molecules offer various opportunities to select a most appropriate trigger for cell-based therapy; such a trigger will not only precisely control therapeutic protein expression but also play a role in health because of its excellent bioactivity. For example, natural molecules with anti-cancer activity such as resveratrol would be a perfect trigger for cell-based cancer therapy because the pharmacological activity of the inducer would strongly enhance the therapeutic effect of the induced therapeutic proteins. Additionally, reprogrammed T cells that express chimeric antigen receptor (CAR) have been used on the clinical stage for tumor immunotherapy; however, the cytokine release syndrome caused by CAR-T technology is still an unresolved challenge. The use of natural molecules with good anti-inflammatory effects would make it possible to improve the safety of CAR-T-based therapy by enabling suitable CAR expression and reducing the inflammation caused by cytokine storm. Furthermore, these natural molecule-mediated gene switches could be used for programmable biocomputing in animals, which may pave the way for the next generation of cell-based therapies. Finally, we believe that the unique features of natural products as gene switches will enable them to have a wide range of applications and provide new strategies for the therapeutic application of synthetic biology.

## Credit author statement

H.Y. and X.W. conceived this review study. X.W., X.Z., L.K., and H.Y. wrote the manuscript. Y.L. prepared the figures. All authors edited and approved the manuscript.

## Declaration of competing interest

The authors declare that they have no known competing financial interests or personal relationships that could have appeared to influence the work reported in this paper.
